# Dietary Survey of Japanese Individuals with Type 2 Diabetes Mellitus on a Low-Carbohydrate Diet: An Observational Study

**DOI:** 10.3390/nu16111658

**Published:** 2024-05-28

**Authors:** Sakiko Inaba, Tomomi Shirai, Mariko Sanada, Hiroyuki Miyashita, Gaku Inoue, Taichi Nagahisa, Noriaki Wakana, Kazuhiro Homma, Naoto Fukuyama, Satoru Yamada

**Affiliations:** 1 Department of Food and Nutritional Science, Graduate School of Applied Bioscience, Tokyo University of Agriculture, 1-1-1 Sakuragaoka, Setagaya-ku, Tokyo 156-8502, Japan; 2 Nutrition Department, Kitasato University Kitasato Institute Hospital, 5-9-1 Shirokane, Minato-ku, Tokyo 108-0072, Japan; 3 Pharmacy Department, Kitasato University Kitasato Institute Hospital, 5-9-1 Shirokane, Minato-ku, Tokyo 108-0072, Japan; 4 Diabetes Centre, Kitasato University Kitasato Institute Hospital, 5-9-1 Shirokane, Minato-ku, Tokyo 108-0072, Japan; 5 Department of Nutritional Science, Faculty of Applied Bioscience, Tokyo University of Agriculture, 1-1-1 Sakuragaoka, Setagaya-ku, Tokyo 156-8502, Japan

**Keywords:** fat–energy ratio, low-carbohydrate diet, type 2 diabetes mellitus

## Abstract

The nutrient intake of persons with diabetes placed on a low-carbohydrate diet remains unclear. This study aimed to assess nutrient intake in persons with type 2 diabetes mellitus treated with a low-carbohydrate diet. The brief-type self-administered diet history questionnaire was used to collect the dietary information of 335 outpatients at Kitasato Institute Hospital, while their clinical characteristics were collected from their electronic medical records. The median age, HbA1c level, and body mass index of the participants were 68 (60–74) years, 49 (45–55) mmol/mol [6.7 (6.3–7.2)%], and 24.0 (21.8–26.7) kg/m^2^, respectively; median energy intake was 1457 (1153–1786) kcal/day; and protein–energy, fat–energy, and available carbohydrate–energy ratios were 18.6 (15.7–21.4)%E, 36.8 (31.6–43.2)%E, and 34.6 (26.0–42.4)%E, respectively. As the available carbohydrate–energy ratio decreased, the fat–energy ratio increased significantly. The total dietary fibre and salt intake were 7.1 (5.6–8.4) g/1000 kcal and 6.5 (5.6–7.5) g/1000 kcal, respectively. Japanese individuals with type 2 diabetes mellitus placed on a low-carbohydrate diet had a fat-to-energy ratio exceeding 30%, while the fat–energy ratio increased as the carbohydrate–energy ratio decreased.

## 1. Introduction

The prevalence of diabetes mellitus is increasing globally. In 2021, the estimated global prevalence of diabetes in the 20–79-year-old age group was 537 million, which is expected to reach 783 million by 2045; approximately 55% of persons with diabetes live in the Western Pacific region and Southeast Asia [1].

The treatment of diabetes involves a combination of diet, exercise, and drug therapy. Diabetic diet therapy is used to treat obesity in the United States and Europe [2,3] because the body mass index (BMI) of persons with diabetes mellitus is higher than those without it [4]. Weight loss reduces the risk of diabetes by 55% [5], and continued weight loss reduces the glycated haemoglobin (HbA1c) level to <6.5% and promotes the discontinuation of hypoglycaemic drugs [6]. Therefore, in the United States and Europe, the mainstream dietary approach used to treat diabetes mellitus is energy restriction, which improves blood glucose concentrations and is associated with weight loss [3,7]. It has recently been reported that Mediterranean-style, low-carbohydrate, vegetarian or vegan, and DASH diets can help to reduce the risk of developing diabetes mellitus and improve blood glucose concentrations. The European Association for the Study of Diabetes (EASD) and the American Diabetes Association (ADA) also recommend these diets for diabetic therapy [3,7,8].

The Japan Diabetes Society (JDS) also recommends the use of energy restriction [9,10]. This is because diabetes mellitus with insulin resistance caused by visceral fat obesity is believed to be increasing in Japan [10]. However, the prevalence of obesity in Japanese individuals with type 2 diabetes mellitus is lower than that of their American counterparts [11,12]. Moreover, the average age of Japanese individuals with diabetes is increasing; in 2021, it was 67.5 years [11]. As such, frailty and sarcopenia are becoming more problematic in this population due to aging. In elderly patients, sufficient energy and protein intake are recommended [10]. Therefore, it is necessary to consider dietary approaches other than energy restriction in Japan. In the present study, we focused on a low-carbohydrate diet, which does not restrict protein, fat, or energy, and a therapeutic diet for diabetes mellitus recommended by the EASD and ADA [8].

A low-carbohydrate diet restricts only carbohydrates without restrictions on protein and fat being clearly defined; this type of diet can be subclassified based on the level of carbohydrate restriction. According to a previous study [13], the Atkins and ketogenic diets have very low carbohydrate intake. The Atkins diet was developed for weight loss in individuals with obesity, with a carbohydrate intake of 20 g/day or less during the early phase. Ketogenic diets are used to treat epilepsy. The classic ketogenic diet typically comprises a 4:1 ratio of fat to carbohydrate and protein in grams [14]. Low-carbohydrate diets limit the amount of carbohydrates consumed to 130 g or less per day, as suggested by the ADA. A diet with a carbohydrate energy intake of 26–45% is considered to be a moderate-carbohydrate diet, as the carbohydrate energy intake of people with obesity is approximately 43%.

In healthy Western people, a low-carbohydrate diet is linked to increased salt intake and decreased dietary fibre, vitamins, and mineral intake [15,16]. However, only a few studies have investigated the nutrient intake of persons with diabetes placed on a low-carbohydrate diet; hence, it is unclear whether they show similar trends as healthy individuals. Furthermore, in Japan, the nutrient intake of persons with diabetes placed on energy restriction diets has been clarified [17]; however, that of those on low-carbohydrate diets has not been investigated. Moreover, although a low-carbohydrate diet is considered to be effective for weight loss and lowering HbA1c [18], low-density lipoprotein cholesterol (LDL-C) and total cholesterol (TC) levels are increased [19]. However, whether this is related to nutrient intake is still being determined, and the effect of protein intake on diabetes has not been investigated. It is necessary to clarify these factors to determine the suitability of low-carbohydrate diets as a therapeutic strategy for diabetes.

Therefore, this study aimed to assess the nutrient intake of individuals with type 2 diabetes on a low-carbohydrate diet.

## 2. Materials and Methods

### 2.1. Participants

The participants in this study were outpatients with type 2 diabetes mellitus aged >30 years at Kitasato Institute Hospital, Tokyo, Japan. This study took place from February 2022 to February 2023. To randomly select participants, the outpatients were numbered, and participants were recruited based on computer-generated random numbers. A dietary assessment was conducted using the single brief-type self-administered diet history questionnaire (BDHQ; Gender Medical Research Inc., Tokyo, Japan) during outpatient care. The participants were interviewed about their intake of low-carbohydrate foods. The following clinical characteristics of the participants were collected from their electronic medical records: (1) duration of diabetes mellitus; (2) duration of low-carbohydrate diet; (3) physical characteristics (height, weight, BMI, systolic blood pressure [SBP], and diastolic blood pressure [DBP]); (4) biochemical parameters (HbA1c, casual plasma glucose, TC, high-density lipoprotein cholesterol [HDL-C], LDL-C, triglyceride [TG]), estimated glomerular filtration rate [eGFR], and albumin–creatinine ratio [ACR]); (5) drug history (antidiabetic, antihypertensive, and antilipemic medications); and (6) diabetic retinopathy.

The protocol for this research project was approved by the suitably constituted Ethics Committee of the institution, and it conforms with the provisions of the Declaration of Helsinki (Kitasato Institute Hospital Research Ethics Committee, Approval No. 21047).

### 2.2. Exclusion Criteria

Participants with an energy intake < 600 kcal/day, which is less than half of the minimum value recommended by the JDS [10], and those with an energy intake > 4000 kcal/day, which is 1.5 times more than the maximum value recommended, were judged to have under-reported or over-reported and were excluded from the analysis [20]. Patients without HbA1c value measurements were excluded from the analysis. Furthermore, insulin users were excluded from the analysis because this could have included people with depleted insulin secretion. Patients receiving prescriptions at other hospitals were excluded from the drug usage rate analysis.

### 2.3. Dietary Assessment

The BDHQ is a variation of a diet history questionnaire designed for adults living in Japan that requires participants to recall and answer questions about meals consumed over a month [21]. It comprises a 58-item fixed-portion-type food frequency questionnaire and a 15-item diet history questionnaire and requires approximately 10–15 min on average to complete. Dietary intake was estimated using a purpose-built computer algorithm. In the BDHQ, the correlation value of carbohydrates with the semi-weighted 16-day dietary records was 0.51 [21]. The BDHQ has been used for dietary assessment in many nutritional epidemiological studies [22,23,24].

### 2.4. Dietary Education

Kitasato Institute Hospital has been using low-carbohydrate diets as a therapeutic approach for persons with diabetes mellitus since 2009. Patients received dietary guidelines on the low-carbohydrate diet from a doctor and registered dieticians at the first medical examination. The intake of available carbohydrates was limited to 20–40 g/meal and up to 10 g of available carbohydrates from snacks. Patients were asked to maintain their available carbohydrate intake within the range of 70–130 g/day. The lower limit of available carbohydrate intake was set to 70 g/day to prevent ketosis. Energy, protein, and fat intake were not restricted [25]. After the first medical examination, the HbA1c and plasma glucose concentrations were assessed during routine examinations, and dietary instructions were provided again when they increased.

### 2.5. Statistical Analysis

Statistical analyses were performed using JMP Pro ver. 17.0.0 (SAS Institute, Cary, NC, USA). Energy-adjusted values were also calculated using the density method. Protein, fat, and available carbohydrates were calculated as percentages of daily energy intake. Non-energy nutrients and alcohol were calculated per 1000 kcal of daily energy intake. After examining normality with the Shapiro–Wilk test, the data were presented as the median (first–third quartile) because most outcomes had a non-normal distribution. The One Way ANOVA, Kruskal–Wallis test and Fisher’s exact tests were used to compare four categories. Multiple comparisons were performed as appropriate using the Tukey–Kramer or Steel–Dwass tests. The *t*-test, Welch’s *t*-test, Mann–Whitney U, and Fisher’s exact tests were used for unpaired two-group comparisons. Statistical significance was denoted by two-sided *p* < 0.05.

## 3. Results

### 3.1. Participant Characteristics

In total, 406 patients consented to participating in this study, and 395 completed a dietary survey. Four patients withdrew their consent before the dietary survey, and seven patients did not visit the hospital on the day of the dietary survey. Six patients with an energy intake < 600 kcal/day (under-reporting), one patient without HbA1c value measurements, and fifty-three insulin users were excluded from the analysis.

Thus, data from 335 patients were analysed (Table 1). A total of 17 of the 335 patients had received prescriptions at other hospitals; therefore, drug history was studied for 318 patients.

Of the 335 participants, 222 were males and 113 were females, with a median age of 68 (60–74) years. The median duration of diabetes was 13 (9–19) years, and the median duration of the low-carbohydrate diet was 9 (5–13) years. The median HbA1c and casual plasma glucose concentrations were 49 (45–55) mmol/mol [6.7 (6.3–7.2)%] and 131 (114–150) mg/dL, respectively. The median BMI was 24.0 (21.8–26.7) kg/m^2^. The median blood pressure and lipid profile were within normal limits. Regarding renal function, the median eGFR and ACR were 68.71 (59.21–78.99) mL/min/1.73 m^2^ and 14.5 (6.4–42.9) mg/gCr, respectively. Diabetic retinopathy was observed in 11.0% of the patients. Antidiabetic, antihypertensive, and antilipemic medications were used by 86.2%, 62.0%, and 72.0% of the patients, respectively (Table 2). The results of clinical parameters, including those of insulin users, are shown in Supplementary Table S1. The median HbA1c level, including that of insulin users, was 50 (46–57) mmol/mol [6.8 (6.4–7.4)%].

### 3.2. Dietary Characteristics

**Figure 1:**
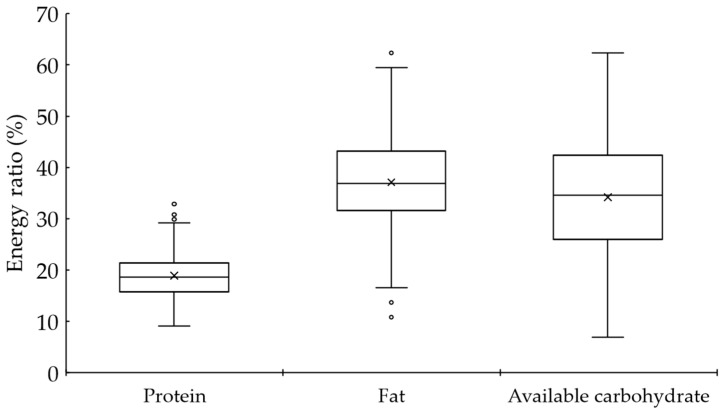
Macronutrient energy ratio.

The median energy intake was 1457 (1153–1786) kcal/day [6098 (4825–7474) kJ/day]. The median energy-adjusted values of protein, fat, and available carbohydrate were 18.6 (15.7–21.4) %E, 36.8 (31.6–43.2) %E, 34.6 (26.0–42.4) %E [119.0 (85.6–162.9) g/day], respectively (Figure 1).

The median saturated fatty acid intake was 9.6 (8.1–11.1) %E. The median total dietary fibre intake, which is reported to be reduced by low-carbohydrate diets [15], was 7.1 (5.6–8.4) g/1000 kcal. The median salt intake, which is reported to be increased by low-carbohydrate diets [15], was 6.5 (5.6–7.5) g/1000 kcal (Table 3). The results of dietary characteristics, including those of insulin users, are shown in Supplementary Table S2 and the results of dietary characteristics by gender are shown in Supplementary Table S3.

### 3.3. Comparison Using Available Carbohydrate–Energy Ratio

The 335 participants were divided into four groups using the interquartile range of the available carbohydrate-to-energy ratio, and participant characteristics, nutrient intake, physical characteristics, and biochemical parameters were compared (Table 4).

The age in Q4 (72 (65–78) years) was significantly higher than in Q1 (68 (57–73) years) and Q2 (66 (58–74) years). The duration of low-carbohydrate diets in Q3 (10 (6–13) years) and Q4 (11 (6–13) years) was significantly longer than in Q1 (7 (3–10) years). As the available carbohydrate–energy ratio decreased, the protein–energy ratio increased significantly, particularly the energy ratio of animal protein. Equally, as the available carbohydrate energy–ratio decreased, the fat–energy ratio increased significantly. Monounsaturated fatty acids, polyunsaturated fatty acids, and cholesterol showed similar results. There was no significant difference in the total dietary fibre among the four groups. The salt intake in Q1 (7.2 (6.3–7.9) g/1000 kcal) was significantly higher than in the other three groups. The alcohol intake in Q4 (0.0 (0.0–3.9) g/1000 kcal) was significantly lower than in the other three groups. The HbA1c value in Q3 (52 (47–59) mmol/mol) was significantly higher than in Q1 (47 (44–55) mmol/mol) and Q2 (48 (45–54) mmol/mol). The BMI value in Q3 (24.6 (22.9–27.8) kg/m^2^) was significantly higher than in Q2 (23.8 (21.0–26.0) kg/m^2^). The TC values in Q2 (196 (167–216) mg/dL) tended to be higher than in Q3 (179 (162–192)) (*p* = 0.059), but there were no significant differences between the other groups. There were no significant differences in the HDL-C, LDL-C, and TG values among the four groups. The eGFR in Q4 (62.51 (55.23–74.53) mL/min/1.73 m^2^) was significantly lower than in Q1 (71.13 (59.21–87.14) mL/min/1.73 m^2^) and Q2 (72.78 (62.10–83.47) mL/min/1.73 m^2^). When age-adjusted eGFR was compared, there were no significant differences among the four groups for those over 68 years of age (Supplementary Table S4). There were no significant differences in the prevalence of diabetic retinopathy among the four groups. The use rates of antidiabetic, antihypertensive, and antilipemic medications also showed no significant differences among the four groups.

## 4. Discussion

Of the 335 participants included in the analysis, 222 were males and 113 were females (Table 1). The high proportion of men in this study is similar to that reported in the National Health and Nutrition Survey in Japan [26]. The median age of the participants was 68 years, similar to the mean age of Japanese individuals with type 2 diabetes mellitus (67.5 years) [11]. The median HbA1c level was 49 (45–55) mmol/mol [6.7 (6.3–7.2) %], and 209 patients (62.4%) achieved the target value of less than 7% for the prevention of complications. Compared to the results of Japanese individuals with type 2 diabetes mellitus, the number of participants with 6.5% ≤ HbA1c < 7.0% was also the highest [11], and in this study, the number of participants with 6.0% ≤ HbA1c < 6.5% was also large.

The median value of available carbohydrate intake was 119.0 g/day [34.6% energy], within the low-carbohydrate diet range. The participants were divided into two groups: Those with an available carbohydrate-to-energy ratio of ˂40% and those with an available carbohydrate-to-energy ratio of ≥40%. The HbA1c value was significantly lower in the group with an available carbohydrate-to-energy ratio of ˂40% (Supplementary Table S5). The intake in this study was approximately half that of Japanese individuals with type 2 diabetes [23]. Since the higher the available carbohydrate–energy ratio, the higher the age of the participant (Table 4), it is thought that carbohydrate restrictions become less strict as age increases.

The median energy intake was 1457 kcal/day (Table 3). Although we judged that the participants were not underweight and that there was no shortage of energy intake because the median BMI was 24.0 kg/m^2^, it is necessary to instruct patients not to consciously limit their energy in consideration of the onset of frailty. We assessed whether protein and fat intake would change as available carbohydrate intake decreased. The median protein–energy ratio was 18.6%. This intake was higher than the value of 16.3% reported in a population of Japanese individuals with diabetes mellitus older than 60 years [23]. Still, it was within the average protein intake recommended for persons with diabetes mellitus with or without kidney disease according to the ADA (1–1.5 g/kgBW/day or 15%−20%E) [7]. Because of this, we determined that protein intake would not increase even if available carbohydrates were restricted. Furthermore, when we assessed the types of protein separately, the animal protein–energy ratio was 12.1%, and the plant protein–energy ratio was 6.3%. Since the energy ratio of animal protein increases as the available carbohydrate energy ratio decreases, meat and fish intake might increase on a low-carbohydrate diet.

Conversely, the median fat–energy ratio was 36.8%, which was higher than the value of 26.8% reported in Japanese individuals with diabetes mellitus older than 60 years [23] and the 20–30% recommended by the JDS [10]. The National Academy of Medicine in America set the upper limit for fat intake at 35% [27], but the fat–energy ratio in the present study exceeded this. Saturated fatty acid intake was also clarified, as the upper limit for the fat–energy ratio was set in line with the upper limit for saturated fatty acid intake [27,28]. The median value of the saturated fatty acid–energy ratio was 9.6%, exceeding the tentative dietary goal for preventing lifestyle-related diseases in Japan (7%) [28].

Furthermore, a low-carbohydrate diet has been linked with reduced dietary fibre and increased salt intake in healthy people who take on a low-carbohydrate diet [15]; therefore, we assessed fibre and salt intake. The total dietary fibre intake in this study was 7.1 g/1000 kcal. However, there were no significant differences in the total dietary fibre intake among the four groups when compared using the interquartile of the available carbohydrate–energy ratio (Table 4). It is thought that there is no decrease in total dietary fibre intake when the available carbohydrate–energy ratio is within the range of 20% to 50%. However, because we were not able to compare the lower/higher available carbohydrate–energy ratios, we believe it is necessary to confirm the incidence of cardiovascular disease and mortality in patients placed on a low-carbohydrate diet. This is because total dietary fibre intake has been reported to be associated with these outcomes [29,30]. Instructing patients to avoid a decrease in dietary fibre may also be necessary. The median salt intake in this study was 6.5 g/1000 kcal. The salt intake in Q1 (7.2 (6.3–7.9) g/1000 kcal) was significantly higher than in the other three groups. Since the energy–animal protein ratio increases as the available carbohydrate–energy ratio decreases, it can be inferred that salt intake increases as a result of the salt used to season meat and fish dishes. The participants were educated on avoiding consuming too many salt-rich foods and seasonings. To maintain good blood pressure in persons with type 2 diabetes mellitus placed on a low-carbohydrate diet, instructions on salt intake reduction may be necessary.

This study had some limitations. This was an observational study. It was necessary to continuously investigate the same individuals’ routine examination results and dietary intake to accurately evaluate the influence of a low-carbohydrate diet. Furthermore, since this dietary survey was conducted using a food frequency method and patients’ diets could not be quantitatively evaluated, the nutrient intake and biochemical parameters were compared based on ranking. The BDHQ reflects dietary intake over the past month. Therefore, it is not possible to determine whether the participants’ nutrient intake remained the same throughout the duration of the low-carbohydrate diet. Additionally, as this dietary survey method relies on a participant’s memory, recall bias may be present in the results. The small number of participants in their 30s and 40s may also have had an impact on this study’s results. In the future, by conducting quantitative dietary surveys, it will be possible to conduct more detailed studies, such as the relationship between energy and nutrient intake and biochemical parameters when following a low-carbohydrate diet. Additionally, this was a single-centre investigation. If more facilities apply a low-carbohydrate nutritional approach in the future, it will be possible to reach conclusions in conjunction with their findings. Regardless, this study is the first to clarify the energy and nutrient intake of Japanese individuals with type 2 diabetes mellitus placed on a low-carbohydrate diet.

## 5. Conclusions

In conclusion, the participants of this study obtained more than 30% of their energy from fat, and the fat–energy ratio increased as the carbohydrate–energy ratio decreased. These results will be useful when considering diabetic nutritional therapy and energy restrictions in Asian countries, including Japan.

## Figures and Tables

**Table 1:** Participant characteristics.

	Normal Limit or Treatment Target *	Total	Male	Female
n		335	222	113
Age (years)		68 (60–74)	68 (59–73)	72 (63–75)
Duration of diabetes (years)		13 (9–19)	14 (9–19)	13 (8–18)
Duration of low-carbohydrate diet (years)		9 (5–13)	9 (5–13)	9 (5–13)
HbA1c (mmol/mol)	<53	49 (45–55)	50 (45–56)	51 (45–55)
(%)	<7.0	6.7 (6.3–7.2)	6.7 (6.3–7.3)	6.8 (6.3–7.2)
Number of <7.0% (n) (%)		209 (62.4)	142 (64.0)	67 (59.3)
Casual plasma glucose (mg/dL)		131 (114–150)	132 (116–153)	128 (110–142)
BMI (kg/m^2^)	22~25	24.0 (21.8–26.7)	24.3 (22.3–26.9)	23.5 (21.5–25.9)
SBP (mmHg)	<130	125 (117–133)	124 (117–130)	128 (117–136)
DBP (mmHg)	<80	73 (67–79)	74 (69–80)	70 (65–76)
TC (mg/dL)		183 (164–205)	180 (160–202)	191 (174–217)
HDL-C (mg/dL)	≥40	62.6 (51.7–76.4)	56.3 (48.5–69.0)	73.3 (60.3–86.6)
LDL-C (mg/dL)	<120	101.3 (83.9–117.0)	100.9 (83.9–115.4)	102.1 (83.0–120.0)
TG (mg/dL)	<150	105 (73–151)	109 (76–157)	97 (72–138)
eGFR (mL/min/1.73 m^2^)		68.71 (59.21–78.99)	67.59 (58.33–79.51)	69.65 (61.35–78.83)
ACR (mg/gCr)		14.5 (6.4–42.9)	14.4 (5.9–43.5)	14.9 (8.1–40.1)
Diabetic retinopathy (%)		11.0	10.8	11.5

The data are expressed as medians (first–third quartile) or numbers (%). HbA1c, glycated haemoglobin; BMI, body mass index; SBP, systolic blood pressure; DBP, diastolic blood pressure; TC, total cholesterol; HDL-C, high-density lipoprotein cholesterol; LDL-C, low-density lipoprotein cholesterol; TG, triglyceride; eGFR, estimated glomerular filtration rate; ACR, albumin–creatinine ratio. * Normal limits or Treatment Target were based on the Japanese Clinical Practice Guideline for Diabetes 2019 [10].

**Table 2:** Details of drug usage.

	Total	Male	Female
n	318	209	109
Antidiabetic medication (%)	86.2	85.2	88.1
Sulfonylurea (%)	23.6	24.9	21.1
Glinide (%)	0.9	1.0	0.9
α-glucosidase inhibitor (%)	4.1	4.3	3.7
Biguanide (%)	67.0	65.1	70.6
SGLT-2 inhibitor (%)	45.3	45.0	45.9
DPP-4 inhibitor (%)	60.4	59.3	62.4
GLP-1 receptor agonist (%)	10.1	11.0	8.3
Thiazolidinediones (%)	3.8	3.8	3.7
Antihypertensive medication (%)	62.0	66.0	54.1
ACE inhibitor (%)	0.6	1.0	0.0
ARB (%)	56.6	60.8	48.6
Mineralocorticoid receptor inhibitor (%)	2.8	3.3	1.8
Ca antagonist (%)	40.6	42.6	36.7
β blocker (%)	5.0	4.3	6.4
Thiazide (%)	6.6	7.2	5.5
Antilipemic medication (%)	72.0	70.8	74.3
Statin (%)	64.8	61.2	71.6
Ezetimibe (%)	7.9	8.1	7.3
Fibrate (%)	11.6	16.3	2.8

The data are expressed as numbers (%). Drug history was analysed for 318 patients.

**Table 3:** Dietary characteristics of participants.

	Crude		Energy-Adjusted by the Density Method	
	Unit	Intake	Unit	Intake
Energy	kcal/day	1457 (1153–1786)	−	−
	kJ/day	6098 (4825–7474)	−	−
Protein	g/kg BW/day	1.0 (0.8–1.3)	% energy	18.6 (15.7–21.4)
Animal protein	g/kg BW/day	0.6 (0.5–0.9)	% energy	12.1 (9.5–15.2)
Plant protein	g/kg BW/day	0.3 (0.3–0.4)	% energy	6.3 (5.3–7.2)
Fat	g/day	56.7 (45.8–75.0)	% energy	36.8 (31.6–43.2)
Saturated fatty acid	g/day	15.1 (11.6–19.6)	% energy	9.6 (8.1–11.1)
Monounsaturated fatty acid	g/day	20.9 (16.6–27.7)	% energy	13.3 (11.1–15.7)
Polyunsaturated fatty acid	g/day	14.1 (11.1–18.3)	% energy	9.1 (7.4–10.6)
Cholesterol	mg/day	446 (290–590)	mg/1000 kcal	297 (233–381)
Available carbohydrate	g/day	119.0 (85.6–162.9)	% energy	34.6 (26.0–42.4)
Total dietary fibre	g/day	9.8 (7.8–13.0)	g/1000 kcal	7.1 (5.6–8.4)
Salt	g/day	9.4 (7.5–11.7)	g/1000 kcal	6.5 (5.6–7.5)
Alcohol	g/day	2.5 (0.0–22.6)	g/1000 kcal	1.6 (0.0–13.7)

The data are expressed as medians (first–third quartile). Energy-adjusted values were also calculated using the density method. Protein, fat, and available carbohydrates were calculated as a percentage of daily energy intake. Non-energy nutrients and alcohol were calculated per 1000 kcal of daily energy intake. BW, body weight.

**Table 4:** Participant characteristics and nutrient intake for each available carbohydrate interquartile range.

	Q1	Q2	Q3	Q4	
Available carbohydrate (%E)	21.2 (16.6–24.3)	30.2 (28.6–32.2)	38.5 (36.4–40.1)	46.9 (44.2–50.9)	
(g/1000 kcal)	53.0 (41.4–60.7)	75.5 (71.5–80.6)	96.2 (91.2–100.2)	117.3 (110.5–127.2)	*p*-value
n (male)	83 (60)	84 (56)	84 (56)	84 (50)	
Age (years)	68 (57–73)	66 (58–74)	68 (60–74)	72 (65–78)	0.002 ‡||
Duration of diabetes (years)	11 (6–17)	13 (6–18)	14 (10–19)	15 (11–20)	0.005 ‡||
Duration of low-carbohydrate diet (years)	7 (3–10)	9 (3–13)	10 (6–13)	11 (6–13)	<0.001 †‡
Protein (%E)	22.2 (19.2–25.9)	20.1 (17.3–21.6)	18.0 (15.2–20.2)	15.9 (14.1–17.6)	<0.001 *†‡§||¶
Animal protein (%E)	15.9 (13.6–18.9)	13.6 (11.1–15.5)	11.1 (9.0–13.4)	9.5 (7.4–11.3)	<0.001 *†‡§||¶
Plant protein (%E)	6.1 (4.6–7.9)	5.8 (5.0–6.8)	6.3 (5.5–7.2)	6.5 (6.1–7.2)	0.005 ||
Fat (%E)	45.5 (39.2–50.1)	41.5 (34.7–44.3)	35.4 (32.7–38.2)	30.1 (26.9–33.0)	<0.001 *†‡§||¶
Saturated fatty acid (%E)	10.6 (9.1–12.3)	10.3 (8.5–12.0)	9.4 (7.9–10.4)	8.5 (7.0–9.6)	<0.001 †‡§||¶
Monounsaturated fatty acid (%E)	16.7 (14.1–19.0)	15.1 (13.0–16.4)	12.9 (11.0–14.3)	10.8 (9.7–11.9)	<0.001 *†‡§||¶
Polyunsaturated fatty acid (%E)	11.7 (9.9–13.0)	9.8 (8.5–11.0)	8.5 (7.2–9.5)	7.2 (6.3–7.9)	<0.001 *†‡§||¶
Cholesterol (mg/1000 kcal)	406 (293–490)	330 (271–393)	275 (229–335)	218 (172–279)	<0.001 *†‡§||¶
Total dietary fibre (g/1000 kcal)	7.4 (5.5–9.5)	7.0 (5.5–8.3)	7.3 (5.9–8.3)	6.8 (5.6–8.0)	0.380
Salt (g/1000 kcal)	7.2 (6.3–7.9)	6.5 (5.7–7.2)	6.4 (5.6–7.4)	6.0 (5.4–7.1)	<0.001 *†‡
Alcohol (g/1000 kcal)	7.3 (0.0–22.1)	1.9 (0.0–21.4)	2.4 (0.0–12.1)	0.0 (0.0–3.9)	<0.001 ‡||¶
HbA1c (mmol/mol)	47 (44–55)	48 (45–54)	52 (47–59)	51 (46–55)	0.002 †§
(%)	6.5 (6.2–7.2)	6.6 (6.3–7.1)	7.0 (6.5–7.6)	6.9 (6.4–7.3)	0.002 †§
Casual plasma glucose (mg/dL)	130 (120–146)	133 (114–154)	133 (114–152)	125 (110–144)	0.431
BMI (kg/m^2^)	23.6 (21.8–26.5)	23.8 (21.0–26.0)	24.6 (22.9–27.8)	24.2 (22.2–26.0)	0.041 §
SBP (mmHg)	122 (115–130)	124 (115–130)	126 (117–135)	128 (119–135)	0.208
DBP (mmHg)	74 (69–79)	72 (66–79)	74 (67–80)	71 (66–76)	0.296
TC (mg/dL)	186 (167–215)	196 (167–216)	179 (162–192)	180 (155–201)	0.021
HDL-C (mg/dL)	65.9 (51.6–82.0)	64.1 (54.4–82.4)	58.9 (51.6–74.8)	58.8 (49.8–73.8)	0.078
LDL-C (mg/dL)	106.7 (88.9–121.1)	103.8 (89.3–120.9)	97.9 (82.1–111.1)	99.6 (80.1–118.7)	0.151
TG (mg/dL)	95 (61–147)	105 (76–139)	108 (73–154)	124 (77–159)	0.139
eGFR (mL/min/1.73 m^2^)	71.13 (59.21–87.14)	72.78 (62.10–83.47)	68.39 (59.22–77.06)	62.51 (55.23–74.53)	0.002 ‡||
ACR (mg/gCr)	15.4 (6.6–42.3)	14.6 (5.6–34.6)	19.7 (8.2–43.6)	13.1 (6.5–46.2)	0.374
Diabetic retinopathy (%)	12.0	9.5	10.7	11.9	0.953
Antidiabetic medication (%)	84.8	80.5	88.9	90.1	0.306
Antihypertensive medication (%)	58.2	61.0	60.5	67.9	0.621
Antilipemic medication (%)	60.8	75.3	72.8	79.0	0.068

The data are expressed as medians (first–third quartiles) or numbers (%). Q1, first quartile; Q2, second quartile; Q3, third quartile; Q4, fourth quartile; HbA1c, glycated haemoglobin; BMI, body mass index; SBP, systolic blood pressure; DBP, diastolic blood pressure; TC, total cholesterol; HDL-C, high-density lipoprotein cholesterol; LDL-C, low-density lipoprotein cholesterol; TG, triglyceride; eGFR, estimated glomerular filtration rate; ACR, albumin–creatinine ratio. Significance level: *p* < 0.05. The *p*-value indicates the result of the One Way ANOVA, Kruskal–Wallis test and Fisher’s exact tests. *, Q1 vs. Q2; †, Q1 vs. Q3; ‡, Q1 vs. Q4; §, Q2 vs. Q3; ||, Q2 vs. Q4; ¶, Q3 vs. Q4, correction by Tukey–Kramer or Steel–Dwass tests.

## Data Availability

The data presented in this study are available on request from the corresponding author due to ethical reasons.

## Supplementary Materials

The following supporting information can be downloaded at: https://www.mdpi.com/article/10.3390/nu16111658/s1. Table S1: Participant characteristics, including insulin users; Table S2: Dietary characteristics of participants, including insulin users; Table S3: Dietary characteristics of participants by gender; Table S4: Age-adjusted eGFR for each available carbohydrate interquartile range; Table S5: Comparison by available carbohydrate-to-energy ratio.
