# Supplementary material for: Dietary Survey of Japanese Individuals with Type 2 Diabetes Mellitus on a Low-Carbohydrate Diet: An Observational Study
Source: Nutrients. 2024 May 28;16(11):1658. doi: 10.3390/nu16111658 (PMC11174919; doi:10.3390/nu16111658)
Supplement: Supplementary file 1 [file nutrients-16-01658-s001.zip › Table S1.pdf]

Table S1. Participant characteristics, including insulin users.

	Total	Non-insulin user	Insulin user	*p*-value
n (male)	388 (254)	335 (222)	53 (32)	
Age (years)	69 (60−74)	68 (60−74)	71 (65−75)	0.078
Duration of diabetes (years)	14 (10−19)	13 (9−19)	18 (15−25)	<0.001
Duration of low-carbohydrate diet (years)	10 (5−13)	9 (5−13)	13 (10−13)	<0.001
HbA1 (mmol/mol)	50 (46−57)	49 (45−55)	58 (52−63)	<0.001
(%)	6.8 (6.4−7.4)	6.7 (6.3−7.2)	7.5 (7.0−8.0)	<0.001
Casual plasma glucose (mg/dL)	131 (114−151)	131 (114−150)	135 (117−159)	0.427
BMI (kg/m^2)	24.1 (21.8−26.8)	24.0 (21.8−26.7)	24.7 (22.3−28.6)	0.291
SBP (mmHg)	125 (117−133)	125 (117−133)	125 (117−134)	0.928
DBP (mmHg)	73 (67−79)	73 (67−79)	70 (64−77)	0.093
TC (mg/dL)	182 (163−204)	183 (164−205)	174 (160−195)	0.182
HDL-C (mg/dL)	61.1 (51.1−75.7)	62.6 (51.7−76.4)	55.9 (47.7−70.3)	0.016
LDL-C (mg/dL)	100.3 (83.9−115.8)	101.3 (83.9−117.0)	94.9 (82.3−108.1)	0.195
TG (mg/dL)	107 (76−152)	105 (73−151)	119 (86−177)	0.083
eGFR (mL/min/1.73m^2)	68.54 (58.88−79.51)	68.71 (59.21−78.99)	67.39 (56.18−82.45)	0.866
ACR (mg/gCr)	15.1 (6.7−46.1)	14.5 (6.4−42.9)	33.0 (8.4−77.1)	0.023
Diabetic retinopathy (%)	15.2	11.0	41.5	<0.001
Antidiabetic medication (%)	88.1	86.2	100.0	0.001
Antihypertensive medication (%)	62.5	62.0	66.0	0.647
Antilipemic medication (%)	73.3	72.0	81.1	0.183

Drug history was analyzed for 371 patients. The data are expressed as medians (first–third quartiles) or numbers (%).

HbA1c, glycated haemoglobin; BMI, body mass index; SBP, systolic blood pressure; DBP, diastolic blood pressure; TC, total cholesterol; HDL-C, high-density lipoprotein cholesterol; LDL-C, low-density lipoprotein cholesterol; TG, triglyceride; eGFR, estimated glomerular filtration rate; ACR, albumin–creatinine ratio

Non-insulin user vs insulin user (The t-test, Mann–Whitney U test, Fisher's exact test)

Significance level: $p<0.05$
