# Supplementary material for: Dietary Survey of Japanese Individuals with Type 2 Diabetes Mellitus on a Low-Carbohydrate Diet: An Observational Study
Source: Nutrients. 2024 May 28;16(11):1658. doi: 10.3390/nu16111658 (PMC11174919; doi:10.3390/nu16111658)
Supplement: Supplementary file 1 [file nutrients-16-01658-s001.zip › Table S2.pdf]

Table S2. Dietary characteristics of participants, including insulin users.

	Crude intake					Intake of Energy-adjusted by the density method				
	Unit	Total	No-insulin user	Insulin user	p-value	Unit	Total	No-insulin user	Insulin user	p-value
Energy	kcal/day	1478 (1171−1829)	1457 (1153−1786)	1632 (1253−2011)	0.057	–	–	–	–	–
	kJ/day	6185 (4898−7652)	6098 (4825−7474)	6827 (5242−8413)	0.057	–	–	–	–	–
Protein	g/kgBW/day	1.0 (0.8−1.4)	1.0 (0.8−1.3)	1.2 (0.9−1.5)	0.049	% energy	18.6 (15.8−21.5)	18.6 (15.7−21.4)	18.0 (15.7−22.5)	0.839
Animal protein	g/kgBW/day	0.6 (0.5−0.9)	0.6 (0.5−0.9)	0.8 (0.5−1.0)	0.116	% energy	12.1 (9.5−15.2)	12.1 (9.5−15.2)	12.4 (9.5−15.3)	0.794
Plant protein	g/kgBW/day	0.3 (0.3−0.5)	0.3 (0.3−0.4)	0.2 (0.3−0.5)	0.085	% energy	6.3 (5.4−7.2)	6.2 (5.3−7.2)	6.2 (5.5−7.1)	0.967
Fat	g/day	58.6 (46.7−75.6)	56.7 (45.8−75.0)	67.3 (49.7−80.1)	0.123	% energy	36.7 (31.7−42.7)	36.8 (31.6−43.2)	36.0 (31.9−40.1)	0.325
Saturated fatty acid	g/day	15.4 (11.6−20.2)	15.1 (11.6−19.6)	17.9 (11.8−22.3)	0.057	% energy	9.6 (8.1−11.1)	9.6 (8.1−11.1)	9.9 (8.7−11.1)	0.865
Monounsaturated fatty acid	g/day	21.1 (16.9−27.8)	20.9 (16.6−27.7)	24.1 (18.7−28.1)	0.177	% energy	13.3 (11.1−15.6)	13.3 (11.1−15.7)	13.0 (11.0−14.9)	0.216
Polyunsaturated fatty acid	g/day	14.3 (11.3−18.3)	14.1 (11.1−18.3)	14.5 (12.1−18.3)	0.376	% energy	9.0 (7.4−10.5)	9.1 (7.4−10.6)	8.5 (7.2−10.2)	0.155
Cholesterol	mg/day	449 (294−594)	446 (290−590)	468 (310−644)	0.225	mg/1000 kcal	297 (233−381)	297 (233−380)	291 (235−372)	0.814
Available carbohydrate	g/day	121.2 (86.0−170.9)	119.0 (85.6−162.9)	137.3 (96.1−193.4)	0.031	% energy	34.9 (26.6−42.5)	34.6 (26.0−42.4)	36.6 (27.8−44.0)	0.293
Total dietary fibre	g/day	10.1 (7.8−13.3)	9.8 (7.8−13.0)	11.4 (8.7−15.1)	0.056	g/1000 kcal	7.1 (5.6−8.4)	7.1 (5.6−8.4)	7.2 (5.3−8.8)	0.711
Salt	g/day	9.5 (7.5−12.0)	9.4 (7.5−11.7)	10.5 (8.2−13.1)	0.027	g/1000 kcal	6.5 (5.7−7.5)	6.5 (5.6−7.5)	6.8 (6.0−7.7)	0.310
Alcohol	g/day	2.5 (0.0−22.1)	2.5 (0.0−22.6)	0.3 (0.0−21.7)	0.387	g/1000 kcal	1.5 (0.0−13.4)	1.6 (0.0−13.7)	0.2 (0.0−12.0)	0.303

The data are expressed as medians (first–third quartile).

Energy-adjusted values were also calculated using the density method. Protein, fat, and available carbohydrates were calculated as a percentage of daily energy intake. Non-energy nutrients and alcohol were calculated per 1000 kcal of daily energy intake.

BW, body weight; E, energy

Non-insulin user vs insulin user (The t-test, Mann–Whitney U test, or Fisher's exact test) Significance level: $p<0.05$
