# Supplementary material for: Dietary Survey of Japanese Individuals with Type 2 Diabetes Mellitus on a Low-Carbohydrate Diet: An Observational Study
Source: Nutrients. 2024 May 28;16(11):1658. doi: 10.3390/nu16111658 (PMC11174919; doi:10.3390/nu16111658)
Supplement: Supplementary file 1 [file nutrients-16-01658-s001.zip › Table S5.pdf]

Table S5 Comparison by available carbohydrate-to-energy ratio.

	available carbohydrate-to-energy ratio		
	< 40%	≥ 40	
Available carbohydrate (%E)	29.4 (23.4−34.9)	45.3 (43.0−49.9)	
(g/1000kcal)	73.5 (58.6−87.4)	113.2 (107.6−124.8)	*p*-value
n (male)	229 (158)	106 (64)	0.136
Age (years)	67 (58−73)	71 (65−78)	<0.001
Duration of diabetes (years)	13 (7−19)	14 (11−20)	0.009
Duration of low-carbohydrate diet (years)	9 (4−13)	11 (7−13)	<0.001
Protein (%E)	20.2 (17.4−22.4)	16.2 (14.4−17.9)	<0.001
Animal protein (%E)	13.9 (11.0−16.3)	9.6 (7.7−11.6)	<0.001
Plant protein (%E)	6.0 (4.9−7.1)	6.5 (6.1−7.2)	<0.001
Fat (%E)	40.4 (35.4−45.1)	30.6 (28.0−34.2)	<0.001
Saturated fatty acid (%E)	10.2 (8.6−11.8)	8.5 (7.2−9.7)	<0.001
Monounsaturated fatty acid (%E)	14.7 (12.9−16.6)	10.9 (9.8−12.3)	<0.001
Polyunsaturated fatty acid (%E)	9.8 (8.4−11.3)	7.2 (6.4−8.2)	<0.001
Cholesterol (mg/1000kcal)	331 (259−417)	236 (178−295)	<0.001
Total dietary fibre (g/1000kcal)	7.2 (5.5−8.6)	7.0 (5.6−8.2)	0.414
Salt (g/1000kcal)	6.7 (5.8−7.6)	6.2 (5.4−7.1)	0.002
Alcohol (g/1000kcal)	3.5 (0.0−20.2)	0.0 (0.0−5.3)	<0.001
HbA1c (mmol/mol)	49 (45−55)	51 (47−56)	0.014
(%)	6.7 (6.3−7.2)	6.9 (6.5−7.3)	0.014
Casual plasma glucose (mg/dL)	131 (116−149)	128 (110−151)	0.370
BMI (kg/m²)	23.9 (21.7−26.8)	24.3 (22.2−26.7)	0.431
SBP (mmHg)	124 (116−131)	127 (118−135)	0.146
DBP (mmHg)	73 (68−79)	72 (66−77)	0.207
TC (mg/dL)	185 (165−209)	180 (159−199)	0.070
HDL-C (mg/dL)	63.2 (52.2−79.6)	60.3 (50.2−74.1)	0.130
LDL-C (mg/dL)	102.5 (85.4−118.1)	99.6 (80.9−114.7)	0.274
TG (mg/dL)	103 (72−145)	118 (76−161)	0.078
eGFR (mL/min/1.73m²)	69.95 (60.76−83.04)	63.20 (54.90−74.59)	<0.001
ACR (mg/gCr)	14.9 (6.1−42.7)	13.4 (6.4−42.9)	0.716
Diabetic retinopathy (%)	10.9	11.3	1.000
Antidiabetic medication (%)	84.7	89.3	0.301
Antihypertensive medication (%)	60.0	66.0	0.325
Antilipemic medication (%)	70.2	75.7	0.351

The data are expressed as medians (first–third quartiles) or numbers (%). HbA1c, glycated haemoglobin; BMI, body mass index; SBP, systolic blood pressure; DBP, diastolic blood pressure; TC, total cholesterol; HDL-C, high-density lipoprotein cholesterol; LDL-C, low-density lipoprotein cholesterol; TG, tri-glyceride; eGFR, estimated glomerular filtration rate; ACR, albumin–creatinine ratio. Energy-adjusted values were also calculated by the density method. Protein, fat, and available

carbohydrate were calculated as a percentage of daily energy intake. Non-energy nutrients and alcohol were calculated per 1000 kcal of daily energy intake. Significance level: $p < 0.05$. The *p*-value indicates the result of the *t*-test, Welch's *t*-test, Mann–Whitney U, or Fisher's exact tests.
