# Supplementary material for: Dietary Survey of Japanese Individuals with Type 2 Diabetes Mellitus on a Low-Carbohydrate Diet: An Observational Study
Source: Nutrients. 2024 May 28;16(11):1658. doi: 10.3390/nu16111658 (PMC11174919; doi:10.3390/nu16111658)
Supplement: Supplementary file 1 [file nutrients-16-01658-s001.zip › Tables S3 and S4.pdf]

Table S3. Dietary characteristics of participants by gender.

	Crude intake			Intake of Energy-adjusted by the density method		
	Unit	Male	Female	Unit	Male	Female
Energy	kcal/day	1556 (1227−1898)	1299 (1039−1590)	−	−	−
	kJ/day	6509 (5136−7941	5434 (4349−6653)	−	−	−
Protein	g/kgBW/day	0.9 (0.7−1.3)	1.1 (0.8−1.4)	% energy	17.8 (15.2−20.7)	19.6 (17.4−22.1)
Animal protein	g/kgBW/day	0.6 (0.5−0.9)	0.7 (0.5−1.0)	% energy	11.4 (9.1−15.1)	12.9 (10.7−15.5)
Plant protein	g/kgBW/day	0.3 (0.2−0.4)	0.4 (0.3−0.5)	% energy	6.1 (5.1−6.9)	6.7 (5.9−7.6)
Fat	g/day	61.7 (48.9−78.1)	52.8 (42.2−67.9)	% energy	36.2 (30.9−42.8)	38.3 (32.1−43.5)
Saturated fatty acid	g/day	15.5 (11.9−20.6)	13.5 (10.9−18.0)	% energy	9.4 (8.0−11.0)	9.9 (8.3−11.7)
Monounsaturated fatty acid	g/day	22.3 (17.6−29.1)	19.4 (15.0−24.1)	% energy	13.2 (11.0−15.7)	13.6 (11.5−15.8)
Polyunsaturated fatty acid	g/day	14.8 (12.0−19.1)	13.1 (9.9−15.7)	% energy	8.9 (7.3−10.6)	9.2 (7.5−10.6)
Cholesterol	mg/day	454 (290−597)	419 (288−567)	mg/1000 kcal	287 (219−373)	311 (250−387)
Available carbohydrate	g/day	125.4 (85.5−173.3)	114.6 (85.7−150.2)	% energy	33.9 (25.4−41.5)	35.3 (28.9−43.4)
Total dietary fibre	g/day	9.5 (7.7−12.7)	10.5 (8.2−13.6)	g/1000 kcal	6.5 (5.6−7.5)	8.0 (6.8−9.7)
Salt	g/day	9.8 (7.8−12.2)	8.0 (6.8−10.8)	g/1000 kcal	6.4 (5.6−7.4)	6.5 (5.7−7.5)
Alcohol	g/day	8.3 (0.0−33.2)	0.0 (0.0−3.8)	g/1000 kcal	4.7 (0.0−20.5)	0.0 (0.0−2.4)

The data are expressed as medians (first–third quartile).

Energy-adjusted values were also calculated using the density method. Protein, fat, and available carbohydrates were calculated as a percentage of daily energy intake. Non-energy nutrients and alcohol were calculated per 1000 kcal of daily energy intake.

BW, body weight

Table S4 Age-adjusted eGFR for each available carbohydrate interquartile range.

	Q1	Q2	Q3	Q4	
**Age ≤ 68 years**					
Available carbohydrate (%E)	20.7 (17.1−23.9)	30.2 (28.1−32.0)	38.5 (36.2−39.8)	47.7 (45.2−53.1)	
(g/1000kcal)	68.6 (43.2−84.6)	112.3 (89.9−143.7)	139.8 (10.9−181.8)	177.9 (146.2−246.7)	
n (male)	46 (30)	48 (34)	45 (35)	33 (24)	
Age (years)	58 (52−62)	59 (53−65)	61 (54−66)	63 (55−67)	0.070
eGFR (mL/min/1.73m^2)	80.47 (69.14−91.27)	74.35 (65.29−84.60)	69.05 (59.74−80.04)	70.64 (57.49−77.64)	0.003†‡
**Age > 68 years**					
Available carbohydrate (%E)	22.3 (16.2−24.6)	30.4 (29.2−33.6)	38.3 (36.6−40.3)	46.1 (43.4−49.9)	
(g/1000kcal)	79.8 (55.7−96.7)	99.8 (85.8−120.3)	139.4 (112.8−174.5)	170.8 (132.8−205.4)	
n (male)	37 (7)	36 (22)	39 (21)	51 (26)	
Age (years)	74 (71−77)	74 (71−78)	74 (72−83)	76 (73−81)	0.092
eGFR (mL/min/1.73m^2)	60.38 (50.42−73.41)	68.52 (58.34−79.40)	66.53 (53.22−76.76)	61.11 (54.11−68.63)	0.144

The data are expressed as medians (first–third quartiles).

Q1, first quartile; Q2, second quartile; Q3, third quartile; Q4, fourth quartile; eGFR, estimated glomerular filtration rate

Significance level: $p<0.05$

The p value indicates the result of the Kruskal-Wallis test.

*, Q1vsQ2; †, Q1vsQ3; ‡, Q1vsQ4; §, Q2vsQ3; ||, Q2vsQ4; ¶, Q3vsQ4, Correction by Tukey-Kramer or Steel-Dwass
